# Research Progress of H_2_S Donors Conjugate Drugs Based on ADTOH

**DOI:** 10.3390/molecules28010331

**Published:** 2022-12-31

**Authors:** Shuai Wen, Changchang Cao, Jianwen Ge, Wenzhe Yang, Yan Wang, Yi Mou

**Affiliations:** College of Pharmacy and Chemistry & Chemical Engineering, Taizhou University, Taizhou 225300, China

**Keywords:** H_2_S donors, ADTOH, clinical drugs, conjugate drugs

## Abstract

H_2_S is an endogenous gas signaling molecule and its multiple biological effects have been demonstrated. The abnormal level of H_2_S is closely related to the occurrence and development of many diseases, and H_2_S donors has important pharmacological implications. In recent years, H_2_S donors represented by ADTOH (5-(4-hydroxyphenyl)-3H-1,2-dithiole-3-thione) are often used to synthesize new ‘conjugate’ compounds that can release H_2_S and parent drugs. These hybrids retain the pharmacological activity of the parent drugs and H_2_S and have a synergistic effect. ADTOH and parent drug hybrids have become one of the important strategies for the development of H_2_S donor conjugate drugs. This review summarizes molecular hybrids between ADTOH and clinical drugs to provide new ideas for the study of H_2_S donor drug design.

## 1. H_2_S and H_2_S Donors

Hydrogen sulfide (H_2_S) is the third endogenous gas signaling molecule discovered after nitric oxide (NO) and carbon monoxide (CO). H_2_S plays an important regulatory role in a variety of physiological and pathological processes [1,2,3]. The reduction of H_2_S level in the body would lead to the occurrence and development of hypertension, atherosclerosis, gastrointestinal ulcer, liver cirrhosis, diabetes, inflammation, Alzheimer’s disease, cancer, and other diseases. Therefore, the supply of exogenous H_2_S is an effective way to solve the above questions [4,5,6,7,8,9]. However, the toxicological concentration of H_2_S is close to the physiological and pharmacological effective concentration, and the gas is volatile. Considering that, it is difficult to accurately control its effective concentration in vivo, which greatly limits the application of H_2_S gas itself as a drug in basic research and clinical trials. Therefore, sustained-release and controllable H_2_S donor drugs have not only broad application prospects, but also important significance for further elucidating the biological effect of H_2_S [10].

**Figure 1:**
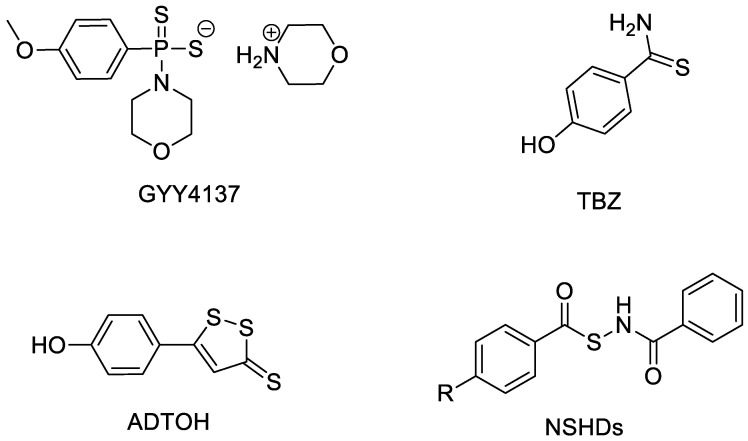
Structures of representative synthetic H_2_S donors.

Like other gas signaling molecules, the activity of H_2_S is closely related to the site, concentration, and velocity of its release [11]. Therefore, the key problem in the study of H_2_S donor drugs is how to improve the selectivity of H_2_S donor molecules, release appropriate concentrations of H_2_S at the target site (generally the lesion site), play a therapeutic role while limiting its adverse reactions. At present, H_2_S donor can be roughly divided into sulfide salt, natural organic sulfur compounds, and synthetic H_2_S donors. Among them, many researchers focused on synthetic H_2_S donors. According to the different molecular structures and functional group properties, they can be broadly classified into thiophosphate derivatives (GYY4137) [12], aryl thiamides (TBZ) [13], 1,2-dithiole-3-thiones (ADT-OH) [14,15], thiol-activated H_2_S donors (NSHDs) [16], etc. (Figure 1).

## 2. ADTOH and Its Conjugates

**Figure 2:**
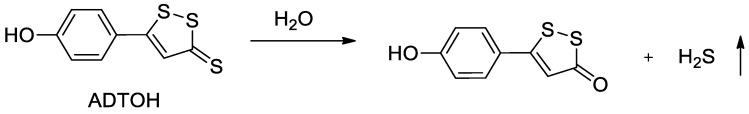
Mechanism of H_2_S release from ADTOH.

5-(4-hydroxyphenyl)-3H-1,2-dithiole-3-thione (ADTOH) is the main metabolite of 5-(4-methoxyphenyl)-3H-1,2-dithiole-3-thione (ADT). The thione of ADTOH can be transformed into the corresponding ketone by hydrolysis and release H_2_S (Figure 2) [17]. In recent years, the research on ADTOH has been boosting. New H_2_S donor derivatives represented by ADTOH have been developed and designed widely, especially in some basic experimental studies. ADTOH is often used to synthesize new ‘conjugate’ compounds that can release H_2_S and parent drugs. Therefore, this paper reviews the hybrid compounds of ADTOH and parent drugs in order to provide a reference for the study of ADTOH-based donor drug design.

### 2.1. ADTOH–NSAID Conjugates

Non-steroidal anti-inflammatory drugs (NSAIDs) are one of the most widely used drugs. NSAIDs generate anti-inflammatory activities by inhibiting cyclooxygenase to reduce the production of inflammatory cytokines such as prostaglandins. However, long-term use of NSAIDs may lead to significant side effects, such as gastrointestinal adverse effects. It has been reported that NO and H_2_S could enhance the local defense of gastric mucosa, thus reducing NSAID-induced gastrointestinal disorders and other side effects [18,19]. Therefore, designing hybrids of H_2_S donors or NO donors together with NSAIDs to enhance the efficacy and reduce the side effects of NSAIDs is an important strategy of current research.

In recent years, new hybrid compounds linking ADTOH with NSAIDs are the most widely studied category in H_2_S research. These ADTOH–NSAID hybrids can release H_2_S and exert H_2_S activity while exerting the pharmacological effects of NSAIDs. The biggest advantage of these compounds compared with the parent NSAIDs is alleviating the gastrointestinal adverse effects.

**Figure 3:**
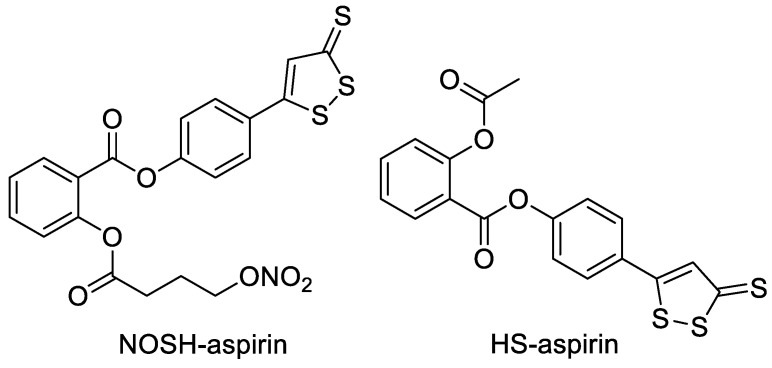
Structures of NOSH-aspirin and HS-aspirin.

NOSH-aspirin is a derivative formed by combining aspirin with ADTOH and NO donors (Figure 3). It has similar antipyretic, analgesic, anti-inflammatory and anti-platelet aggregation effects as aspirin, but has less adverse effects on gastrointestinal bleeding and better tumor prevention effects. In addition, HS-aspirin, a hybrid of aspirin and ADTOH exhibited inhibitory effects in estrogen receptor-negative breast cancer cells and leukemic Jurkat cells [20,21].

**Figure 4:**
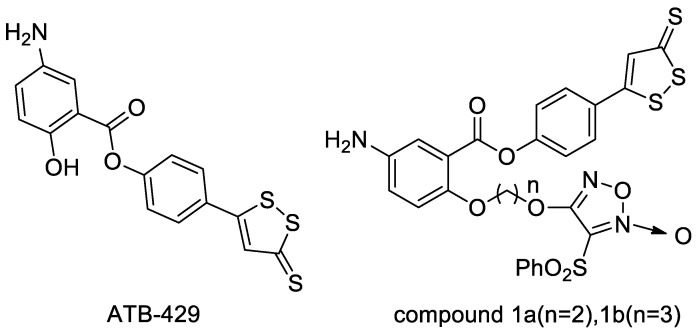
Structures of ATB-429 and compound **1a**, **1b**.

ATB-429 is a hybrid of mesalamine and ADTOH, which has improved anti-inflammatory and analgesic activity (Figure 4). ATB-429 significantly reduced gastrointestinal side effects compared to mesalamine, especially in a mouse model of colitis with better anti-inflammatory activity. ATB-429 exerts anti-inflammatory effects in LPS-induced liver injury, lung injury, ulcerative colitis. Additionally, it has anti-inflammatory effects in NSAID-induced gastric mucosal injury. ATB-429 is superior to mesalamine in reducing mucosal damage and disease severity; moreover, it significantly reduces the infiltration of chronic granulocytes and the expression of several important inflammatory cytokines mRNA. ATB-429 has entered Phase III clinical trials with the U.S. FDA [22]. Inspired by the above findings, Wang et al. [23] designed and synthesized a series of ATB-429 derivatives containing NO-releasing moieties, and evaluated its anti-tumor activity. The results showed that the derivatives have strong anti-tumor activity. Among them, compound **1a** (IC_50_ = 2.677 µM), 1b (IC_50_ = 3.051 µM) against MCF-7 cancer cell line and **1a** (IC_50_ = 1.270 µM) against DU145 cancer cell line are more active than Vandetanib (IC_50_ = 3.536, 1.974 µM).

**Figure 5:**
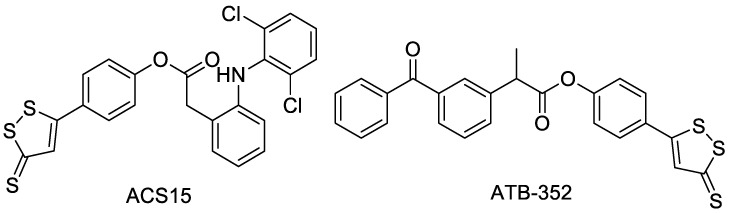
Structures of ACS15 and ATB-352.

ACS15 is an H_2_S donor-type derivative formed by the combination of diclofenac and ADTOH (Figure 5). Compared with diclofenac, it has better anti-inflammatory effects and less gastrointestinal adverse effects. ACS15 can release H_2_S in vitro and in vivo, thus not only improving anti-inflammatory activity, but also significantly reducing the lung injury associated with pancreatitis [24]. Another study found that ACS 15 had anti-myocardial ischemia reperfusion injury activity, while diclofenac did not show this activity [25].

ATB-352 is an H_2_S donor derivative obtained by the coupling of ketoprofen and ADTOH (Figure 5). Studies demonstrated that ATB-352 not only showed anti-inflammatory activity similar to ketoprofen, but also had almost no side effects on the gastrointestinal tract. It can be used for chemoprevention of tumors [26].

**Figure 6:**
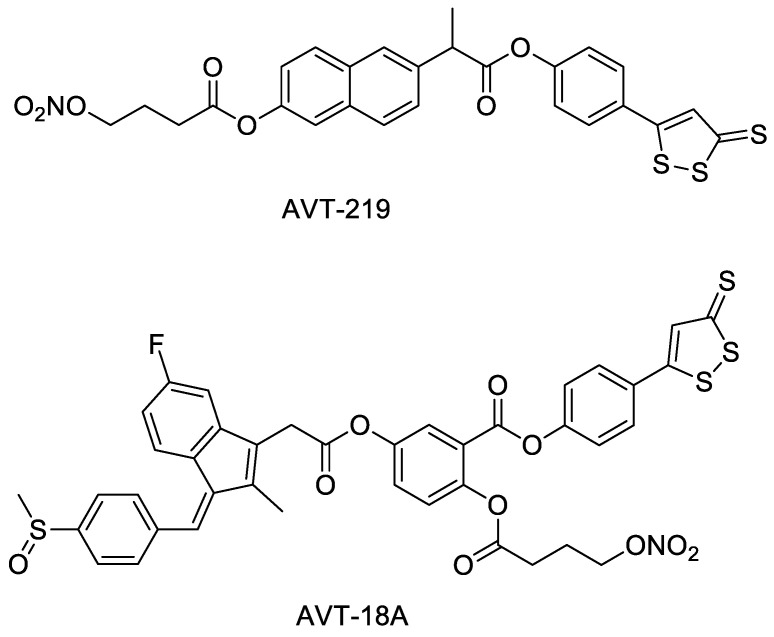
Structures of AVT-219 and AVT-18A.

AVT-219 and AVT-18A (Figure 6) are NOSH drug complexes formed by combining naproxen and sulindac with ADTOH and NO donors [27]. Both AVT-219 and AVT-18A maintain the anti-inflammatory and anti-platelet aggregation properties of naproxen and sulindac. However, the side effect of naproxen and sulindac on the gastrointestinal tract is reduced. These NOSH compounds also have the ability to inhibit the growth activity of many tumor cells, including colon cancer cells, breast cancer cells, and pancreatic cancer cells [28].

### 2.2. ADTOH–Butylphthalide Conjugates

Despite the wide range of drugs available for the clinical management of ischemic stroke, none have yet achieved satisfactory results in the treatment of ischemic stroke. Blocking multiple components of the pathophysiological development of cerebral ischemia is the key to treating this type of disease. For ischemic stroke, the appropriate amount of exogenous NO and H_2_S supplementation can relax blood vessels, inhibit platelet aggregation, increase cerebral blood flow, and protect neuronal cells. On the other hand, supplementation of NO and H_2_S can inhibit the expression of iNOS, which is beneficial for the prevention and treatment of ischaemic brain injury diseases. Therefore, research on NO and H_2_S donor in anti-ischemic brain injury is a hot topic of current research [29,30].

Butylphthalide (NBP) is an effective stroke prevention drug. It inhibits platelet aggregation and reduces thrombosis and cerebral infarct volume. NBP can act on multiple pathological aspects of acute ischemic stroke through multiple targets, pathways, and links, and its clinical effects have been confirmed; however, it still has many shortcomings and needs to be developed further [31,32].

**Figure 7:**
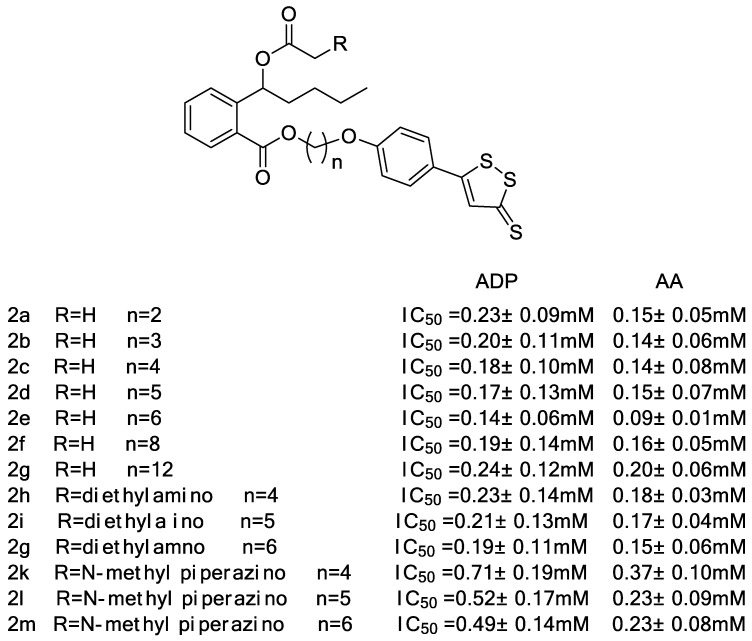

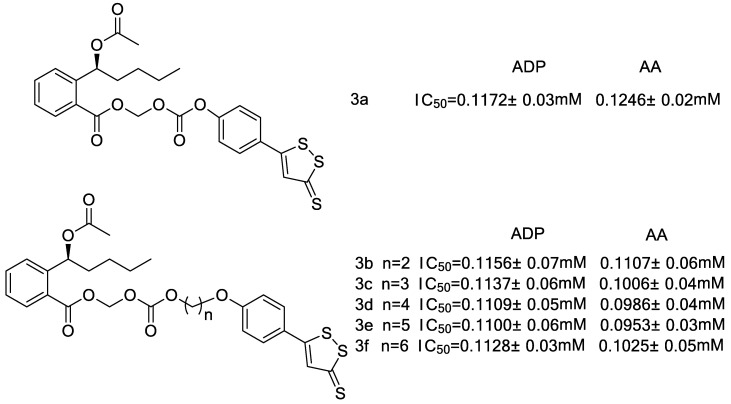
Structures of ADTOH–NBP conjugates.

Wang et al. [33] synthesized a series of hydrogen sulfide-releasing derivatives by collocating butylphthalide with ADTOH and performed a biological evaluation of them (Figure 7). In vitro experiments, compound **2e** significantly inhibited adenosine diphosphate (ADP) and arachidonic acid (AA)-induced platelet aggregation, with better effects than NBP, ticlopidine hydrochloride and aspirin. In addition, **2e** produces moderate levels of H_2_S slowly in vitro, which is beneficial for improving cardiovascular circulation. On top of that, **2e** has a protective effect on collagen and epinephrine-induced thrombosis in mice and exhibits stronger antithrombotic activity than NBP and aspirin in rats. In conclusion, **2e** has promising applications in the treatment of thrombosis-related ischemic strokes.

Inspired by the above findings, Wang et al. [34] designed and synthesized a series of ADTOH-butylphthalide derivatives (Figure 7) by combining the ring-opening derivative of butylphthalein with ADTOH. Among them, compound **3e** showed significantly better inhibitory activity than butylphthalide against platelet aggregation induced by adenosine diphosphate and arachidonic acid.

**Figure 8:**
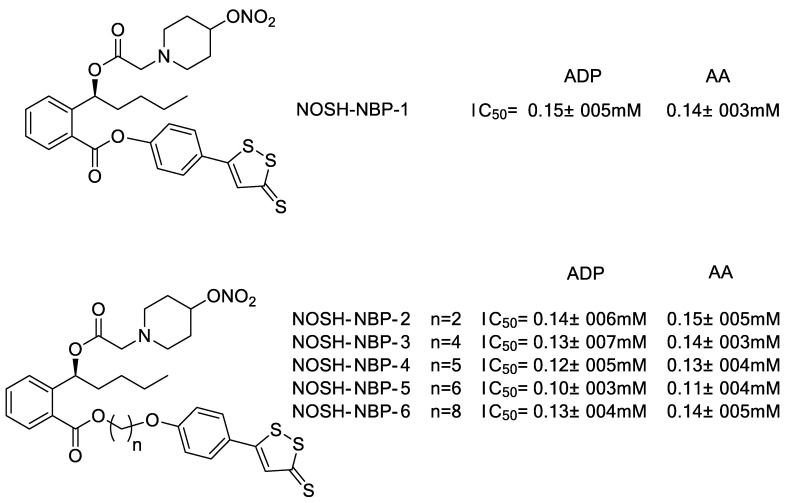
Structures of NOSH–NBP conjugates.

Additionally, Wang et al. [35,36] also synthesized a series of new NOSH-type compounds by combining butylphthalide with NO donors and ADTOH (Figure 8). Compared to butylphthalide parent, compound NOSH-NBP-5 has stronger anti-platelet aggregation activity and is capable of releasing both NO and H_2_S, exerting a protective effect on cardiovascular and cerebral circulation.

### 2.3. ADTOH–Niacin Conjugates

Niacin is known to be involved in lipid metabolism in the body, reducing plasma triglyceride and very low-density lipoprotein concentrations and increasing high density lipoprotein levels [37]. In addition, niacin has a vasodilating effect and is therefore commonly used clinically to treat hyperlipidemia, headaches, venous migraines, and cerebral artery thrombosis. Recent studies have shown that niacin can act as a neuroprotective agent in the treatment of stroke [38]. Therefore, the development of H_2_S donor-nicotinic acid hybrids designed to exert a synergistic neuroprotective effect is considered as a potential therapeutic strategy for ischemic brain injury.

**Figure 9:**
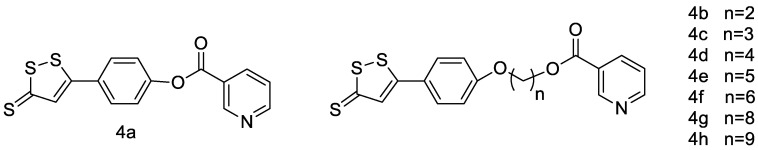
Structures of ADTOH–niacin conjugates.

Sun et al. [39] synthesized a range of derivatives by combining nicotinic acid with ADTOH. Most of the compounds were found to exhibit significant neuroprotective effects. Among them, compound **4f** (Figure 9) can significantly reduce the volume of cerebral infarction in the pMCAO model. The results suggest that such compounds have promising applications in the interventional treatment of cerebral ischemic injury.

### 2.4. ADTOH–Levodopa Conjugates

**Figure 10:**
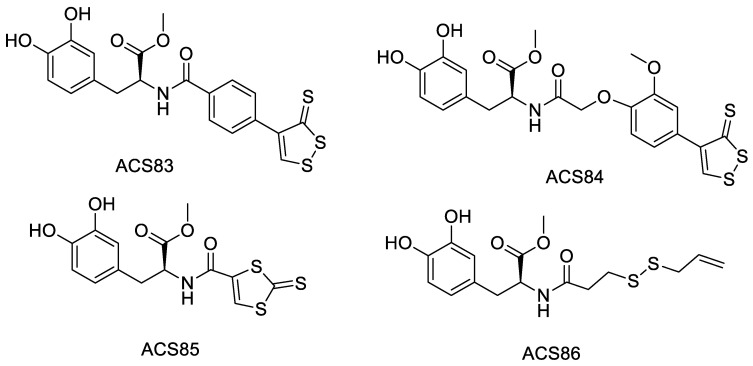
Structures of ADTOH–levodopa conjugates.

Levodopa (L-DOPA) is currently an important drug in the treatment of Parkinson’s syndrome, but it only replenishes dopamine levels in the brain and does not inhibit the progression of the disease. Lee et al. [14] have combined L-DOPA with ADTOH to obtain a series of H_2_S-releasing derivatives (compounds ACS83 to ACS86, Figure 10). These derivatives not only can release dopamine but also protect nerves, and have antioxidant effects. Research has shown that ACS84 can avoid β-amyloid-induced neuronal cell damage through anti-inflammatory effects, and protect mitochondrial in p38- and JNK-mediated stress responses. Thus, ACS84 has the potential to treat neurodegenerative diseases [40].

### 2.5. ADTOH–Doxorubicin Conjugates

**Figure 11:**
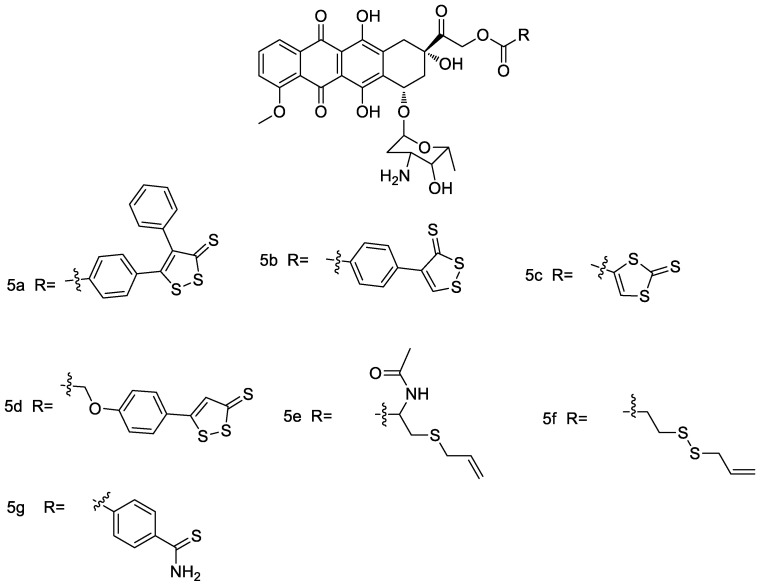
Structures of ADTOH–doxorubicin conjugates.

Doxorubicin is one of the most clinically effective antitumor agents, but cardiotoxicity and drug resistance limit its clinical use. In order to design doxorubicin derivatives with low cardiotoxicity and resistance, Chegaev et al. [41] used doxorubicin as a parent for conjugating with different hydrogen sulfide donors to obtain a series of compounds that can release hydrogen sulfide (compound **5b** and **5d**, Figure 11). It was found that all of these compounds reduced oxidative stress in cardiomyocytes, and some of them showed stronger activity in sarcoma cell lines. Unlike doxorubicin, most of the products are non-toxic to H9c2 cells at 5 μM concentration and have potential for further research and development.

### 2.6. ADTOH–Latanoprost Conjugate

**Figure 12:**
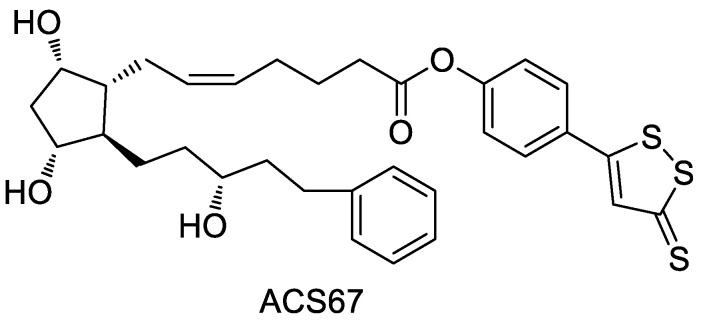
Structure of ACS67.

Latanoprost (Xalatan) is an inactive but rapidly penetrating substance in the cornea, which can be hydrolyzed to active free acid in the cornea and plasma, increasing the outflow of atrial water through the corneal layer and having a good IOP lowering effect. Perrino et al. [42] designed and synthesized a hybrid (ACS 67, Figure 12) by combining latanoprost acid with ADTOH. Experimental data showed that this compound could increase the production of glutathione in the atrial fluid of rabbit eyes, antagonize the oxidative damage of hydrogen peroxide on the neuronal cells in the fundus, and thus alleviate the retinal ischemic damage, with significant optic neuroprotective effect. It has a significant effect on the treatment of glaucoma [43]. In addition, it has been found that ACS67 can inhibit L-type Ca^2+^ channels and reduce L-type voltage-dependent Ca^2+^ channel currents in pancreatic β-cells, thereby inhibiting insulin secretion, but the inhibitory effect is lower than that of NaHS [44].

### 2.7. ADTOH–Monastrol Conjugate

**Figure 13:**
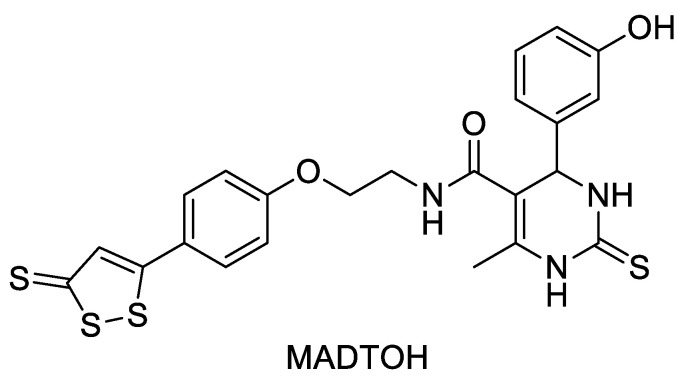
Structure of MADTOH.

Monastrol, a mitotic kinesin inhibitor with potent and cell-permeable properties, was reported as an anti-cancer inhibitor back in the 1990s. Recent studies have shown that monastrol also has calcium channel blocker effects. Considering that H_2_S also has some calcium channel blocking effect, Braga et al. [45] designed and synthesized a Monastrol–ADTOH hybrid (MADTOH, Figure 13) using a conjugate strategy. Compared to monastrol, this compound was effective in reducing the overall calcium transient amplitude in cardiac myocytes via L-type calcium channels. Most notably, the intermediates ADTOH and monastrol were less effective than the hybrid MADTOH in controlling Ca^2+^ homeostasis. Overall, ADTOH hybridized with calcium channel blockers has a wide range of applications in the discovery of suitable calcium channel blockers.

### 2.8. ADTOH–Proglumide Conjugate

**Figure 14:**
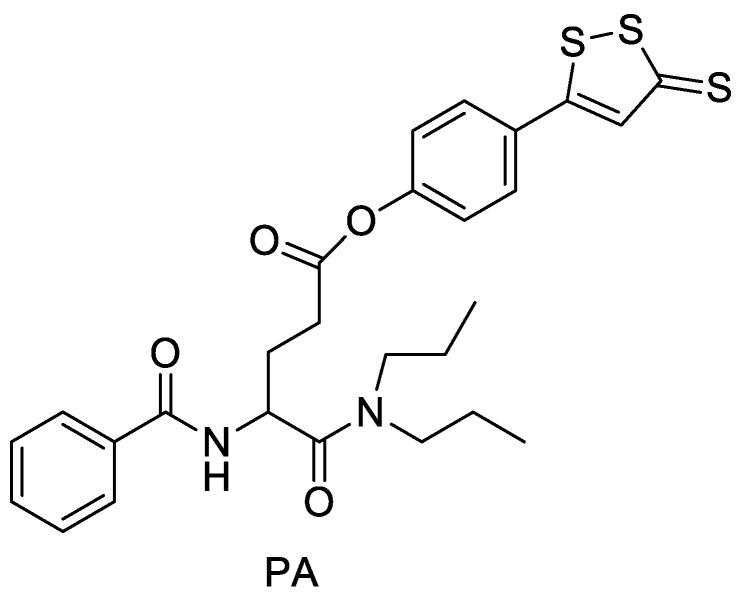
Structure of PA.

Atherosclerosis is a common cardiovascular disease. H_2_S has important physiological functions in atherosclerotic lesions, and many H_2_S donors have been synthesized to study atherosclerosis diseases. Proglumide can reduce the release of cytokines and inflammatory mediators in acute pancreatitis by inhibiting the activation of the NF-кB pathway. Considering that one of the etiologies of atherosclerotic disease is related to inflammation, Ou et al. [46] combined Proglumide with ADTOH to create a hybrid PA (Figure 14). Studies demonstrated that PA is a novel slow-releasing H_2_S donor and shows anti-atherosclerotic effect on the HUVECs injured model by inhibiting the activation of JAK/STAT pathway and NF-кb pathway.

### 2.9. ADTOH–Sildenafil Conjugate

**Figure 15:**
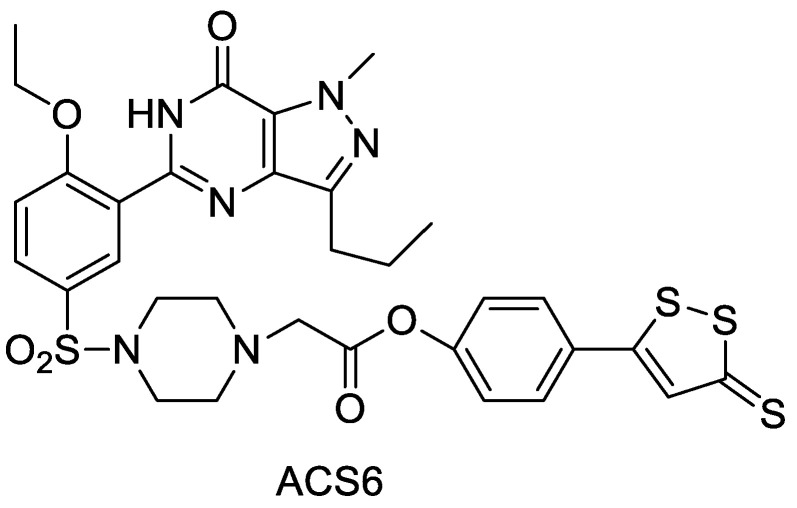
Structure of ACS6.

Sildenafil is a drug that treats erectile dysfunction and pulmonary arterial hypertension. Muzaffar et al. used sildenafil as the parent and ADTOH to form the derivative ACS6 (Figure 15) and showed that ACS6 could enhance the efficacy by slowly releasing hydrogen sulfide, and its diastolic effect on spongy smooth muscle was much stronger than that of the parent drug sildenafil [47] and comparable to sildenafil citrate. Both inhibited oxygen radical generation in pulmonary artery endothelial cells compared with NaHS solution, but the effect was stronger than that of NaHS solution. Moreover, NaHS acts through the cAMP/PKA pathway, and ACS6 activates both the cAMP/PKA and cGMP/PKG pathways. It was also found that ACS 6 protects PC12 cells by upregulating paraoxonase-1 (PON-1) levels, which exerts anti-Hcy-induced neurotoxic and anti-oxidative stress effects [48,49].

### 2.10. ADTOH–Glucocorticoids Conjugates

**Figure 16:**
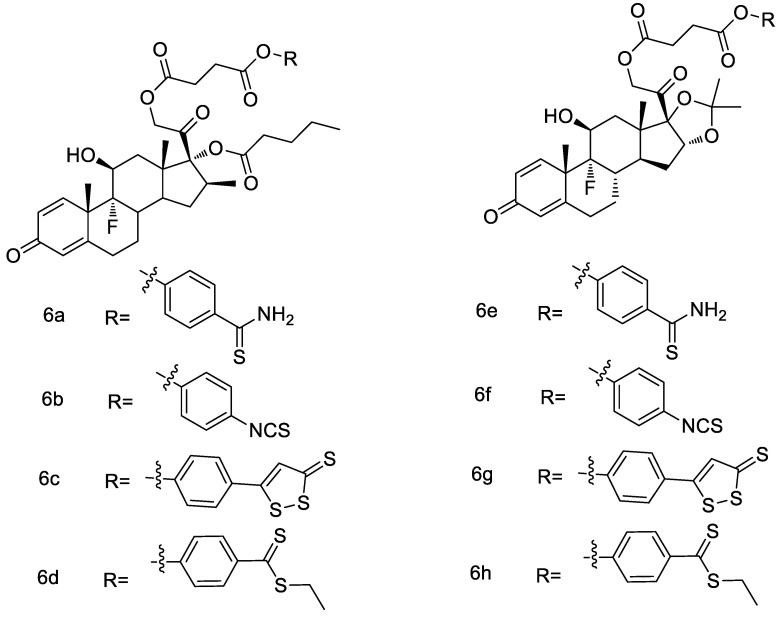
Structures of betamethasone and triamcinolone conjugates.

Asthma is a heterogeneous clinical syndrome. Glucocorticoids are the most effective drugs for treating inflammation in asthma patients. Recent studies have shown that H_2_S has positive effect on this disease. Considering that an improved pharmacological activity and a reduced toxicity can be obtained through hybridization, Giordano et al. [50] designed and synthesized novel betamethasone and triamcinolone hybrids with H_2_S -donors (Figure 16). These synthesized compounds have potential H_2_S-releasing characteristics both in a cell-free environment and into the cytosol of BSMCs (bronchial smooth muscle cells). Among them, the most promising derivatives 6b and 6f have significant inhibitory effect on mast cell degranulation, resulting in a reduction of β-hexosaminidase release more efficiently than the corresponding parent drugs.

**Figure 17:**
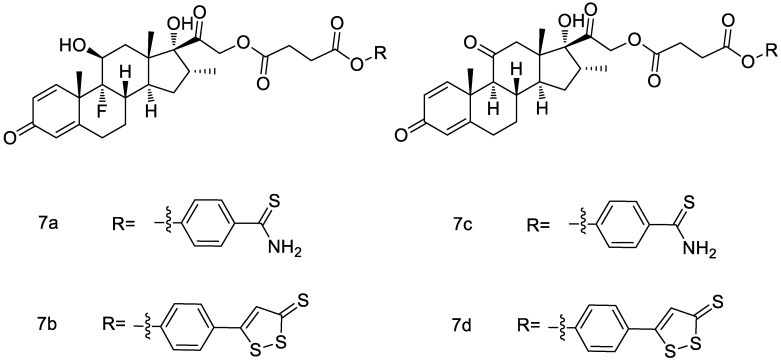
Structures of prednisone and dexamethasone conjugates.

Additionally, Corvino et al. [51] also synthesized a series of novel prednisone and dexamethasone hybrids with two H_2_S -donors (Figure 17). The chemical stability of the synthesized hybrids has been investigated at differing pH values and in human serum. The results show that these hybrids have a prolonged chemical stability both at acidic and physiological pH. Among them, compound **7c** was more effective than prednisone in inhibiting mast cell degranulation and in promoting BSMCs membrane hyperpolarization. Due to the protective effect on airway remodeling, compound **7c** can be a potentially useful therapeutic option for allergic asthma treatment.

**Figure 18:**
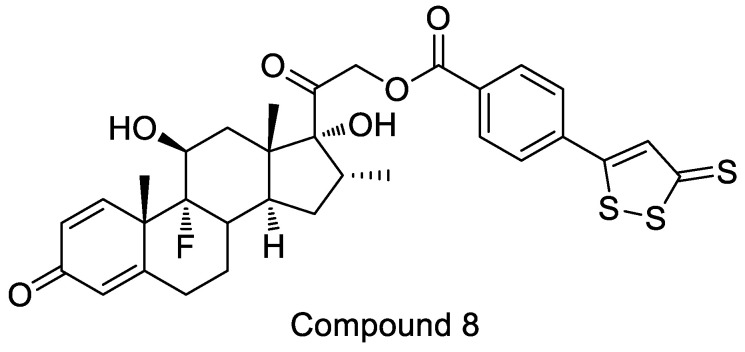
Structure of compound **8**.

Compound **8**, a hybrid of dexamethasone and H_2_S -donor moietie [52], is used to treat ocular diseases (Figure 18). Compound **8** has the ability to completely inhibit oxidative stress-induced glutathione depletion. This design not only eliminated the side effects associated with the parent compound, but also improved pharmacological effects.

### 2.11. ADTOH–Atorvastatin Conjugates

**Figure 19:**
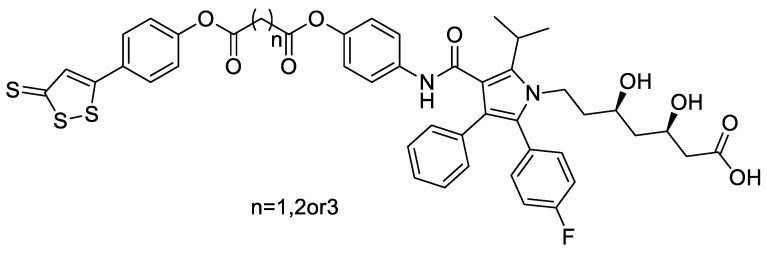
Structures of ADTOH–atorvastatin conjugates.

Atorvastatin is an HMG-CoA reductase inhibitor that reduces plasma cholesterol and lipoprotein levels. Tong et al. [53] designed and synthesized a series of atorvastatin–ADTOH hybrids (Figure 19). Compared with the parent drug atorvastatin, these compounds showed good activity of regulating blood lipids and anti-inflammatory and antioxidant properties. The results showed that these compounds have high application value in regulating blood lipids and vascular protection.

### 2.12. ADTOH–Pentacyclic Triterpene Conjugates

**Figure 20:**
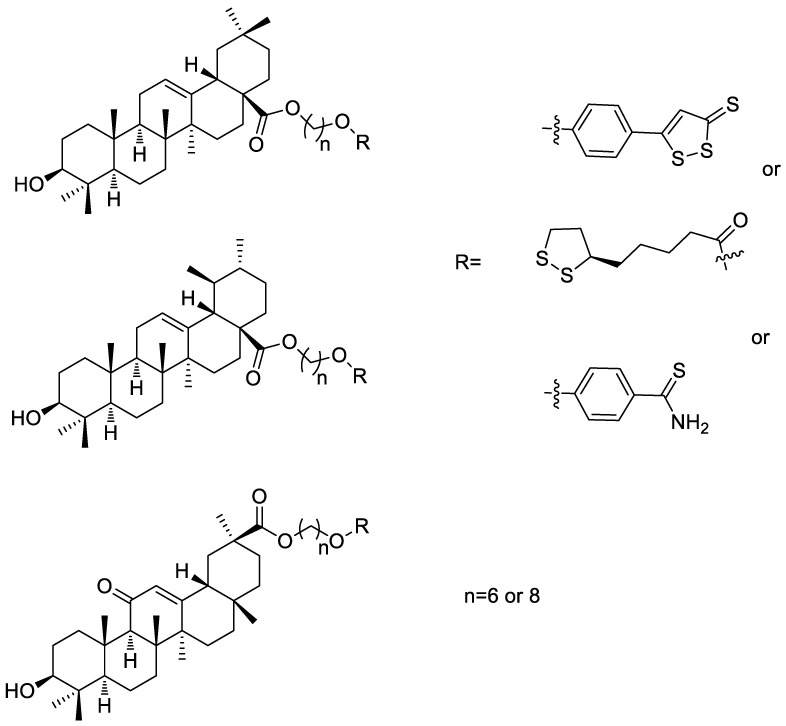
Structures of ADTOH–pentacyclic triterpene conjugates.

Oleanolic acid, ursolic acid, and glycyrrhetinic acid are the active ingredients of herbal medicines, belonging to pentacyclic triterpenes, which have been deeply researched for their various biological activities. Sheng et al. [54] attached the hydrogen sulfide donors to oleanolic acid, ursolic acid, and glycyrrhetinic acid to afford series of pentacyclic triterpenes-H_2_S donor hybrids (Figure 20). The anti-proliferation activity of these hybrids on the tested cell lines was evaluated by MTTassay. The results showed that most of these pentacyclic triterpenes-H_2_S donor hybrids exhibited no anti-proliferation activity against tested cell lines. Therefore, it is not suitable to hybridize hydrogen sulfide donors with oleanolic acid, ursolic acid, and glycyrrhetinic acid in anti-tumor applications.

## 3. Conclusions

As an endogenous gas signaling molecule, H_2_S has been shown to have a variety of biological effects. Decreased levels of H_2_S in the body are closely associated with the development and progression of many diseases, therefore, using H_2_S donors to increase the internal H_2_S concentration has important pharmacological significance. In recent years, research on ADTOH has been on the rise, and H_2_S donors represented by ADTOH are often used to synthesize novel ‘conjugate’ compounds that can release H_2_S and parent drugs. This design conforms to the idea of ‘multi-mechanism drugs’.

As a gas molecule, the activity of H_2_S is closely related to its release site, concentration and velocity. Although ADTOH is frequently used for the design and synthesis of H_2_S donor drugs, its selectivity of H_2_S-release still needs to be improved. In addition, how to design a long-acting, slow-controlled release of H_2_S from ADTOH conjugate drugs, and ensure it releases appropriate H_2_S concentrations at the target site is another key issue in the development of conjugate drugs. This review summarizes the hybrid compounds of ADTOH and parent drugs in order to provide a reference for the study of ADTOH-based donor drug design.

## Data Availability

All data have been included in the manuscript.
